# Hypothalamic–Pituitary–Thyroid and Adrenal Axis Modulation in Response to Fetal Porcine Reproductive and Respiratory Virus Infection

**DOI:** 10.1002/cph4.70112

**Published:** 2026-02-09

**Authors:** Alyssa A. Smith, Glenn Hamonic, Graham S. Plastow, John C. S. Harding, J. Alex Pasternak

**Affiliations:** ^1^ Department of Animal Sciences Purdue University West Lafayette Indiana USA; ^2^ Department of Animal and Food Sciences University of Kentucky Lexington Kentucky USA; ^3^ Department of Large Animal Clinical Sciences, Western College of Veterinary Medicine University of Saskatchewan Saskatoon Saskatchewan Canada; ^4^ Department of Agricultural, Food and Nutritional Science University of Alberta Edmonton Alberta Canada

**Keywords:** HPA axis, HPT axis, NTIS, PRRSV, thyroid hormone

## Abstract

Porcine reproductive and respiratory virus (PRRSV) has been shown to cause a substantial decrease in circulating thyroid hormone levels, consistent with nonthyroidal illness syndrome (NTIS) observed in response to other nidoviruses. This effect is particularly profound following fetal infection, whereby the ability to decrease circulating triiodothyronine is associated with resilience following late‐gestation infection. We have previously shown that the thyroidal response to fetal infection is associated with peripheral changes in deiodinase activity, but the role of the central regulatory axis has not been established. To assess this, we characterized the impact of fetal PRRSV infection on gene expression within the hypothalamic–pituitary‐thyroid (HPT) and ‐adrenal (HPA) axes and further assessed the impact of fetal genotype at a previously identified single nucleotide polymorphism found to contribute to PRRSV‐resilience. In this study, fetal infection and the corresponding NTIS‐like state were associated with modulations in both the HPT and HPA axes, with the most marked effects observed within the thyroid and adrenals, respectively. In the HPT axis, our results indicate altered thyroid hormone metabolism and signaling, with dysregulation of key thyroid hormone receptor, deiodinase, and transporter genes. Similarly, in the HPA axis, the observed transcriptional dysregulations indicate alterations in both steroidogenesis and catecholamine production, with increases in circulating cortisol also indicating a disruption within this system. The results were found to be partially dependent on fetal genotype, collectively providing insights into not only the impact of fetal infection on these critical endocrine systems, but the impact of genotype on the endocrine response to infection.

## Graphical abstract

This study investigates the impact of fetal porcine reproductive and respiratory virus (PRRSV) infection on the Hypothalamic–Pituitary‐Thyroid/Adrenal axes. We report that infection‐induced nonthyroidal illness syndrome is associated with multitissue dysregulation of key genes involved in the control of endocrine function, providing insights into disease‐induced allostatic adaptations.

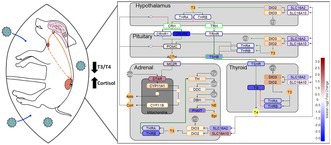

## 1 Introduction

Bioactive thyroid hormones, including thyroxine (T4), triiodothyronine (T3), and additional metabolites such as reverse T3 (rT3) and 3,3′, 3′,5′, or 3,5 diiodothyronine (T2) act as primary regulators of metabolic activity on a cellular level. In addition, this collection of hormones plays a fundamental role in fetal development, driving growth and the maturation of critical organs including the brain and heart (Forhead and Fowden 2014). Much like humans, the porcine fetal thyroid gland becomes functional around 40% of the way through gestation (around day 46 of the normal 115 day gestational period) (Brzezińska‐Slebodzińska and Slebodziński 2004), with fetal levels of circulating T3 and T4 increasing substantially as gestation progresses (Smith and Pasternak 2025). However, unlike humans, which depend at least in part on the vertical transfer of maternal thyroid hormone, the epitheliochorial placenta of the pig acts as a potent enzymatic barrier to maternal thyroid hormones (Krysin et al. 1997).

As in postnatal organisms, the availability of thyroid hormone in the developing fetus is centrally regulated by the hypothalamic–pituitary‐thyroid (HPT) axis, a largely self‐regulating homeostatic endocrine system, wherein negative feedback on the hypothalamus and pituitary maintain circulating levels of thyroid hormones within a physiologically beneficial range. In response to stressful events, the HPT is able to shift its underlying set points in order to adapt to atypical physiological states or pathological challenges (Chatzitomaris et al. 2017). A primary example of this allostatic response is nonthyroidal illness syndrome (NTIS), where, in response to critical illness, circulating T3 and T4 are substantially decreased. While the corresponding increase in biologically inert rT3 suggests this is partially driven by increased peripheral metabolism, the absence of a concomitant increase in circulating thyrotropin (TSH) implies a central regulatory mechanism (Langouche et al. 2019). The underlying mechanism behind such a response remains elusive, but bidirectional relationships between the HPT and other endocrine axes are of significant interest. These other endocrine axes include the hypothalamic–pituitary–adrenal (HPA) axis, which is known to influence both peripheral metabolism of thyroid hormone (Bianco et al. 1987) and the central drivers of its production and release (Samuels and McDaniel 1997; Wilber and Utiger 1969). Similarly, alteration in circulating thyroid hormone levels has been shown to alter baseline cortisol levels and the pituitary and adrenal response to corticotropin releasing hormone (CRH) (Johnson et al. 2005; Kamilaris et al. 1987, 1991). These interactions are consistent with a growing body of evidence that indicates bidirectional relationships and crosstalk between various endocrine axes (Chen et al. 2021; Toews et al. 2021) that have historically been studied in isolation. In light of this, a combined conceptual view of the hypothalamic–pituitary‐thyroid‐adrenal (HPTA) axis yields a more holistic understanding in the context of stress response.

Porcine reproductive and respiratory virus (PRRSV) is a single‐stranded RNA virus which, along with coronaviruses, belongs to the Nidovirales order. This multimodal virus creates respiratory illness in young pigs and reproductive failure in pregnant animals, with severity dependent on the specific viral species and strain. The reproductive consequences of PRRSV result from vertical transmission during the last third of gestation, where the majority of the fetal population becomes infected, while a minority appear to display complete resistance and remain uninfected (Van Goor et al. 2020). The fetal response to infection within a litter is likewise variable, with a resilient subset of infected fetuses remaining viable, and their more susceptible counterparts displaying varying degrees of fetal compromise ranging from meconium staining to fetal death (Harding et al. 2017). Both resilient and susceptible fetuses experience widespread developmental disruption as a result of in utero infection (Malgarin et al. 2021; Mulligan et al. 2022), and those that survive to birth are born weak, underdeveloped, and more susceptible to other pathogens (Feng et al. 2001).

Similar to SARS‐CoV‐2 (Zhang et al. 2021), both fetal and postnatal PRRSV infection severely reduces circulating T3 and T4 levels, with the degree of suppression associated with fetal outcomes (Pasternak et al. 2020b, 2021). Much like the fetal immune response to PRRSV infection (Pasternak et al. 2020a), uninfected fetuses experience only minor decreases in circulating thyroid hormone levels as a result of maternal infection, whereas individuals with high viral loads exhibit a ~75% reduction in T4 and a ~25% reduction in T3 (Pasternak et al. 2020b). In contrast to nonpathogenic porcine fetal hypothyroidism (Ison et al. 2023), PRRSV‐induced thyroid disruption is coincident with decompensation of thyroid hormone metabolism within peripheral fetal organs and within the maternal fetal interface (Ison et al. 2022). However, the mechanism underlying this apparent type 1 allostatic response within the HPT axis, and the influence of this endocrine shift on the HPA axis, has not been investigated. Interestingly, a single nucleotide polymorphism (SNP) (rs80998415) immediately downstream of *iodothyronine deiodinase 2* (*DIO2*) was previously identified as a potential marker for fetal resilience to PRRSV infection (Yang et al. 2016). More recently, this particular SNP was also found to influence fetal thyroid hormone levels following maternal challenge, with the homozygous AA genotype associated with a significant increase in circulating T4 at all levels of fetal infection and preservation (Ko et al. 2022). To characterize the central transcriptional changes regulating the endocrine response to fetal PRRSV infection, the present study conducted a targeted transcriptomic analysis of 24 genes across the four tissues comprising the HPT and HPA axes. Additionally, the present study incorporated the rs80998415 genotype into the fetal selection criteria to better understand the role of this putatively important SNP on activity within the HPT and HPA axes following fetal PRRSV infection.

## 2 Materials and Methods

### 2.1 Late Gestation PRRSV Challenge

Fetal tissue samples for the present study were collected at the termination of a late gestation PRRSV challenge experiment, conducted in accordance with the guidelines of the Canadian Council of Animal Care, with approval of the University of Saskatchewan's Animal Research Ethics Board (Protocol #20180071), and previously described in detail (Ko et al. 2022). Briefly, prospective Landrace gilts and Yorkshire sires, either homozygous AA or BB at the rs80998415 SNP, were identified from routine genotype data and the genotype verified by allelic discrimination qPCR. A total of 13 AA gilts were bred to one of three AA sires, and 14 BB gilts bred to one of 3 BB sires to produce a total of 27 litters homozygous for the target SNP. An inoculum of PRRSV‐2 strain NVSL 97–7895 was prepared on MARC‐145 cells and 22 pregnant gilts challenged with 1 × 10^5^ TCID50 via a combination of intramuscular and intranasal atomization on gestation day 86. The remaining 5 gestationally age matched gilts were sham inoculated to produce a population of control fetuses (CON).

On gestation day 107, 21 days post maternal inoculation, the gilts were euthanized with a combination of intravenous barbiturate (Euthanyl Forte, Bimeda‐MTC, 77 mg/kg sodium pentobarbital), penetrating captive bolt, and exsanguination, followed by extraction of the gravid uterus. All fetuses were deeply phenotyped, but dead, autolyzed, or mummified fetuses were excluded from further analyses. Fetuses found to have visible pulsations within the umbilical cord were categorized as either viable (VIA) or meconium stained (MEC) based on the abundance and distribution of yellow or brownish material on their skin. Fetal blood samples were collected from the axillary artery, allowed to clot at room temperature, and then centrifuged at 2500 × g for 15 min to isolate serum. Thyroid glands (ROID) were identified by their dark crimson color and location on the ventral aspect of the trachea immediately below the larynx and collected along with the cervical and thoracic thymus (THY). The narrow tubular adrenal glands, located dorsomedial to the cranial pole of the right and left kidney, were then excised. Subsequently, the craniovertebral junction was severed, skull cap removed, and the head inverted to sever the optic chiasma and pituitary stalk in order to liberate the whole fetal brain. The diencephalon, including the thalamus and hypothalamus (HYP), was then excised from the central region of the ventral aspect of the brain. Incisions were made on the lateral sides of the sella turcica, and the anterior and posterior pituitary (PIT) were extracted with a small scoopula. Histological sections of HYP, PIT, ROID, and ADR were prepared from an example fetus, and the specificity of isolation for each tissue can be seen in Figure S1. All samples of fetal tissues were snap frozen in liquid nitrogen and subsequently stored at −80°C for later analysis.

### 2.2 Fetal Endocrinology and Viral Load

Total triiodothyronine (T3) and thyroxine (T4) in serum from all fetuses were quantified using commercial RIAs (MP Biomedical), with fetal T3 and T4 levels for all fetuses in the present study reported previously (Ko et al. 2022). Interassay variability, as determined from three unique pools of pig serum, was 7.39% and 3.33% for T3 and T4, respectively. Intraassay variability across all samples was 4.22% and 3.53% for T3 and T4, respectively. Fetal viral load was determined using qPCR as previously described (Ladinig et al. 2014). In short, RNA was extracted from fetal serum and thymus using the Viral RNA Mini and RNeasy mini kits (Qiagen), respectively. Viral RNA was then quantified relative to a standard curve using a one‐step reverse transcription quantitative PCR reaction with PRRSV strain NVSL 97–7895 specific primers (5′‐TAATGGGCTGGCATTCCT‐3′ and 5′‐ACACGGTCGCCCTAATTG‐3) and a hydrolysis probe (5′‐HEX‐TGTGGTGAATGGCACTGATTGRCA‐BHQ2–3′). The resultant viral load in log10 PRRSV RNA copies per μL of serum and mg of thymic tissue were then calculated. Serum cortisol and aldosterone were measured on a subset of fetuses in a single assay run using commercial RIAs. Intraassay variation in the cortisol (MP Biomedical) and aldosterone (IBL America) assays were 3.78% and 3.97%, respectively.

### 2.3 Sample Selection

A total of 316 fetuses were derived from the 22 PRRSV infected litters, of which 203 were found to have pulsations in the umbilical cord and were thus fully phenotyped. A total of 99 and 104 fetuses were homozygous AA or BB, respectively, for the rs80998415 SNP, from which subsets representing biological extremes in viral load and serum thyroid hormone were selected (Table 1). As the timing of fetal infection relative to maternal inoculation varies within a litter (Malgarin et al. 2019), fetal subsets for assessment were selected to represent biologically extreme phenotypes based on a combination of serum and thymic viral load and serum T3 and T4. Ten euthyroid, AA fetuses with no detectable virus in either serum or thymus and normal total T3 and T4 serum levels (mean T3: 0.72 ± 0.13, T4: 98.1 ± 15.27 nmol/L) were categorized as EU‐AA. An equivalent uninfected group of 10 BB fetuses with normal total T3 and T4 serum levels (mean T3: 0.90 ± 0.26, T4: 95.06 ± 13.43 nmol/L) were categorized as EU‐BB. Ten AA fetuses with serum and thymic viral load greater than 5 log, with low serum T3 and T4 levels (mean T3: 0.50 ± 0.16, T4: 23.63 ± 9.74 nmol/L) were categorized as NTIS‐AA. Ten equivalently infected BB fetuses with low serum T3 and T4 levels (mean T3: 0.37 ± 0.21, T4: 26.80 ± 11.10 nmol/L) were categorized as NTIS‐BB. Finally, seven control (CON) fetuses, 3 AA and 4 BB, with mean serum T3 and T4 levels of 0.87 and 97.18 nmol/L respectively, were selected from gestationally age‐matched, sham‐inoculated gilts. Where possible, the selected fetuses were balanced across litter and experimental batch (except CON that were all in batch 1). As a direct result of this selection approach, viral load in the CON and euthyroid groups (EU‐AA & EU‐BB) are all identical, while serum and thymic viral load is significantly greater (*p* < 0.001) in both NTIS‐AA and NTIS‐BB fetuses (Table 1).

**TABLE 1:** Phenotypic characteristics of experimental groups selected based on rs80998415 genotypes and biological extremes for viral load in serum, viral load in thymus, and circulating T3 and T4 levels. EU and NTIS refer to infection status and thyroid hormone levels, while AA and BB refer to the rs80998415 genotype.

	CON	EU‐AA	EU‐BB	NTIS‐AA	NTIS‐BB
*N*	7	10	10	10	10
Serum total T3 (nmol/L)	0.87^a^ (0.72–1.1)	0.72^a^ (0.55–0.89)	0.90^a^ (0.65–1.47)	0.5^b^ (0.19–0.88)	0.37^b^ (0.2–0.67)
Serum total T4 (nmol/L)	97.18^a^ (79.68–127.5)	98.12^a^ (81.85–132.22)	95.06^a^ (80.54–116.14)	23.63^b^ (9.72–46.07)	26.8^b^ (9.89–39.46)
Serum viral load (log10 CN/μL)	0^a^ (0–0)	0^a^ (0–0)	0^a^ (0–0)	8.12^b^ (7.19–8.63)	7.77^b^ (6.79–8.7)
Thymic viral load (log10 CN/mg)	0^a^ (0–0)	0^a^ (0–0)	0^a^ (0–0)	6.68^b^ (5.1–8.09)	6.79^b^ (5.01–8.76)

*Note:* Data are presented as mean and range, with statistical differences (*p* < 0.05) indicated by unique superscripts.

### 2.4 Gene Expression Analysis

The HYP, PIT, ROID, and ADR from each select fetus were cryogenically homogenized with a mortar and pestle, and RNA was isolated using the PureLink RNA mini kit (Thermofisher Scientific) in accordance with the manufacturer's instructions. Potential DNA contamination was removed using Turbo DNase (Invitrogen), and the final concentration and purity of the isolated RNA were determined using a Nanodrop spectrophotometer (Thermofisher Scientific). RNA integrity was assessed using denaturing agarose gel electrophoresis as previously described (Kent‐Dennis et al. 2019) before 2 μg of RNA from each sample was reverse transcribed using the High Capacity cDNA Reverse Transcription kit (Thermofisher Scientific). Target‐specific primer pairs were obtained from the literature or designed using the current RefSeq mRNA sequences corresponding to the *Sus Scrofa* 11.1 genome assembly (Table 2). Primer efficiency for each target was determined to be greater than 90%, and single target amplification was indicated by the presence of a single postamplification melting point. Real‐time qPCR was performed in duplicate on a CFX qPCR system (BioRad) using SsoFast EvaGreen Supermix (BioRad) and 20 ng cDNA per reaction. The tissue‐specific stability of four reference genes was evaluated, and the geometric mean of the most stable genes was used to normalize expression data within each respective tissue. Fold changes were calculated relative to the average expression of each gene within each tissue in the CON group using the 2^−ΔΔCT^ method.

**TABLE 2:** Porcine specific primer sequences used for qPCR.

Function	Symbol	Gene ID	Tissues	Forward primer	Reverse primer	Source
Enzyme	CYP11A1	403,329	ADR	5′‐CACGTAGCCTGAATGTGCA‐3′	5′‐GGGTGGAGTCTCAGTGTCTC‐3′	NM_214427
	CYP11B	110,260,194	ADR	5′‐CAGTATGCCAACAAAGCCATC‐3′	5′‐CATGTTTGCGTGTGTCAGC‐3′	XM_021088772
	DBH	733,609	ADR	5′‐CTGGATTCCCAGCAGGATTA‐3′	5′‐TGCCGTCCTCGATGAAGTA‐3′	XM_001927211
	DDC	396,857	ADR	5′‐TCCTTTCTTCGTGGTGGCTA‐3′	5′‐GGAACTCAGGGCAGATGAAG‐3′	NM_213854
	DIO1	414,380	ROID	5′‐ACTTCATGCAAGGCAACAGG‐3′	5′‐GGTCCTGGAGATTCTGGTGA‐3′	Pasternak et al. (2020b)
	DIO2	414,379	HYP, PIT, ROID, ADR	5′‐CTCGGTCATTCTCCTCAAGC‐3′	5′‐TCACCTGTTTGTAGGCATCG‐3′	Pasternak et al. (2020b)
	DIO3	414,378	HYP, PIT, ROID, ADR	5′‐CCTATCTGCGTGTCTGACGA‐3′	5′‐GCCTGCTTGAAGAAATCCAG‐3′	Pasternak et al. (2020b)
	PNMT	100,144,479	ADR	5′‐CGATTGGAGTGTCTACAGCC‐3′	5′‐ACGTCGATGGGCAGGATC‐3′	NM_001123164
	STAR	396,597	ADR	5′‐TGCTGAGTAAAGTGATCCCAGA‐3′	5′‐GCAGGATCTTGATCTTCTTGACA‐3′	NM_213755
	TH	110,259,705	ADR	5′‐GGAGAACAAGGTCCTCTGGT‐3′	5′‐GGTACACCTGGTCCGAGAAG‐3′	XM_021085452
Ligand or Precursor	CRH	100,127,468	HYP	5′‐TGGATCTCACCTTCCACCTC‐3′	5′‐CCATCAGTTTCCTGTTGCTG‐3′	NM_001113062.1
	POMC	396,863	PIT	5′‐GGAAGATGCCGAGATTGTGC‐3′	5′‐GCACGCCAGCAAGTTACTTT‐3′	NM_213858
	TG	100,156,471	ROID	5′‐CAGTGGCTTCTTCGAGTGTG‐3′	5′‐CGTCACCTCTCCTCCTTTCA‐3′	NM_001168418
	TRH	100,513,309	HYP	5′‐CCAGGGCGATAGTGAATCAG‐3′	5′‐TCTGCCTCTTCCTCCCTTTT‐3′	XM_021068420
	TSHB	397,658	PIT	5′‐ATGACACGGGATTTCAATGG‐3′	5′‐GTGGGCATCCTGGTATTTCT‐3′	NM_214368.2
Receptor	CRHR1	397,426	PIT	5′‐ATGAGAAGTGCTGGTTTGGC‐3′	5′‐TGCGGACGATGTTGAAAAGG‐3′	NM_001144110
	CRHR2	100,240,719	PIT	5′‐CCTCATCGCCACCTTTATCC‐3′	5′‐CACCAGACCTCGTTGCTCT‐3′	XM_021078456
	MC2R	100,739,231	ADR	5′‐CCATTTCTGACATGCTGGGC‐3′	5′‐GGAGTCCACTACGTCGTCAG‐3′	XM_003482032
	THRA	397,387	HYP, PIT, ROID, ADR	5′‐GAGGAGAACAGTGCCAGGTC‐3′	5′‐CGACACACTGCTCGTCTTTG‐3′	Pasternak et al. (2020b)
	THRB	396,776	HYP, PIT, ROID, ADR	5′‐AAGGCTGCAAGGGTTTCTTT‐3′	5′‐TGGCACTGATTTCTGGTGAC‐3′	Pasternak et al. (2020b)
	TRHR	100,415,777	PIT	5′‐AGCCCAGTTTCTCTGCACAT‐3′	5′‐GTAGCCACAGGACACCACAAT‐3′	NM_001177488
	TSHR	397,560	ROID	5′‐CCAACCTTGCTGGATGTCTC‐3′	5′‐TCAGCTCGTGTGAGGTGAAG‐3′	NM_214297
Solute Carrier	SLC16A10	100,513,770	HYP, PIT, ROID, ADR	5′‐CACCCATTGCAGGGTTACTC‐3′	5′‐TATGGAGCCAAGGGATGAAA‐3′	Pasternak et al. (2020b)
	SLC16A2	100,513,513	HYP, PIT, ROID, ADR	5′‐AGTGGAGTTCCAAGCAGCAT‐3′	5′‐AGCCCAAACGATCAGTGAAT‐3′	Pasternak et al. (2020b)
Reference	GAPDH	396,823	HYP, PIT, ROID, ADR	5′‐CTTCACGACCATGGAGAAGG‐3′	5′‐CCAAGCAGTTGGTGGTACAG‐3′	Käser et al. (2015)
	HPRT1	397,351	HYP, PIT, ROID, ADR	5′‐GGACTTGAATCATGTTTGTG‐3′	5′‐CAGATGTTTCCAAACTCAAC‐3′	Käser et al. (2015)
	RPL19	396,989	HYP, PIT, ROID, ADR	5′‐AACTCCCGTCAGCAGATCC‐3′	5′‐AGTACCCTTCCGCTTACCG‐3′	Pasternak et al. (2015)
	YWHAZ	780,440	HYP, PIT, ROID, ADR	5′‐TGATGATAAGAAAGGGATTGTGG‐3′	5′‐GTTCAGCAATGGCTTCATCA‐3′	Pasternak et al. (2020b)

### 2.5 Data Analysis and Visualization

All data analyses, statistics and visualization were carried out using the R programming language version 4.2.3 “Shortstop Beagle” (R Core Team 2023). Confirmation of the differences in group phenotypes with regards to viral load and serum T3 and T4 were conducted by ANOVA and post hoc pairwise *t*‐tests using the Benjamini‐Hochberg correction. A conservative, nonparametric statistical analysis using Kruskal–Wallis followed by post hoc pairwise Wilcoxon rank‐sum test with Benjamini‐Hochberg correction for multiple testing was used to assess the heteroskedastic and/or nonnormal gene expression and serum aldosterone and cortisol data. The threshold for statistical significance was set at *p* < 0.05 and statistically significant changes denoted with unique superscripts, with 0.05 < *p* < 0.1 reported as trends. Quantitative results for qPCR fold changes relative to CON are presented in the form of boxplots using ggplot2 (Wickham 2016), with individual datapoints shown to illustrate the underlying distribution (Weissgerber et al. 2015). To summarize the collective impact of infection across the HPT and HPA axes, multitissue pathway heat maps were generated using a custom plotting script based on the Grid Graphics Package (R Core Team 2023).

## 3 Results

### 3.1 Hypothalamic Gene Expression

**FIGURE 1:**
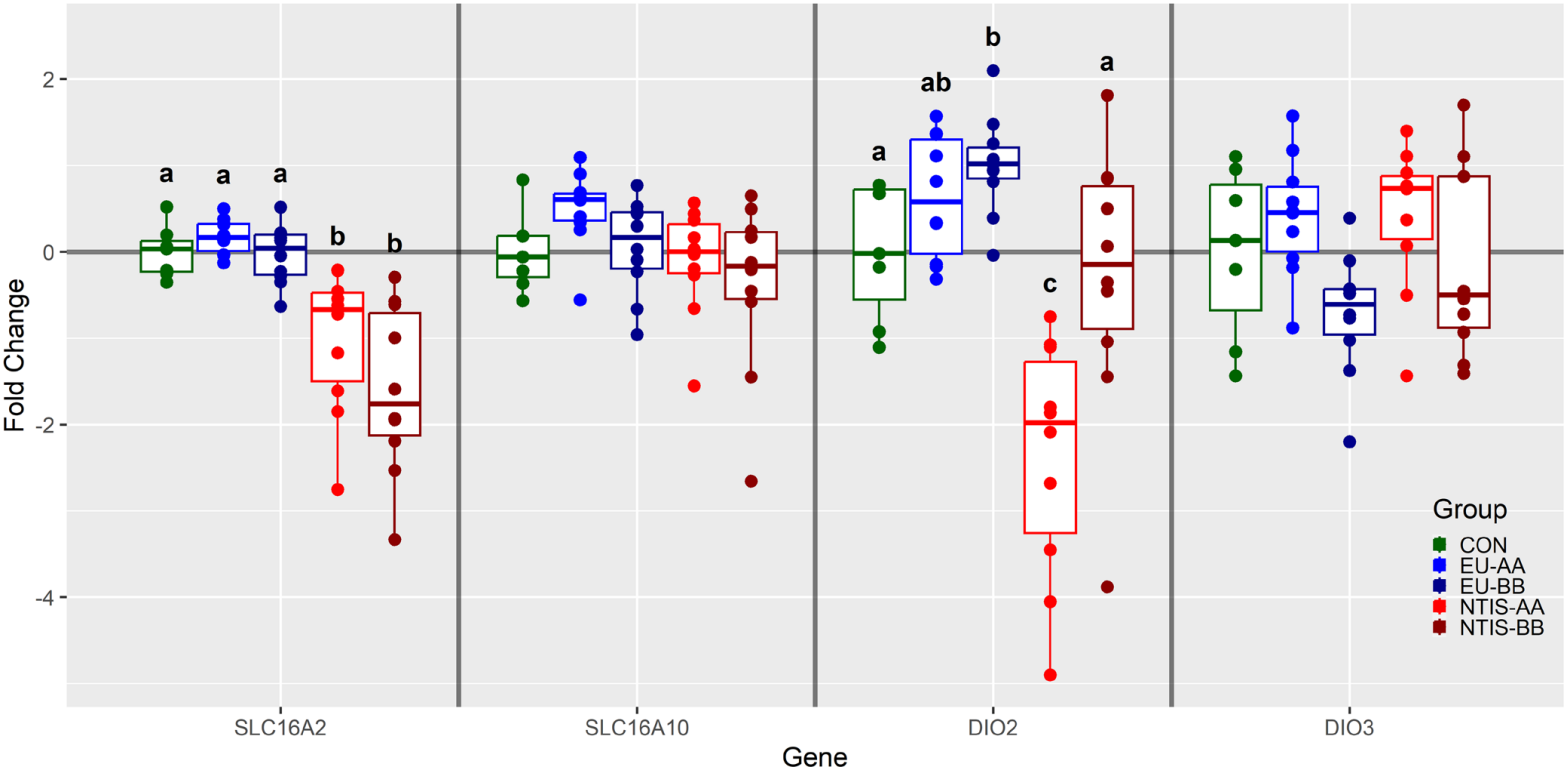
Expression of a subset of genes assessed in diencephalon including the hypothalamus (HYP) from control (CON), euthyroid uninfected (EU), and infected low thyroid hormone (NTIS) fetuses by rs80998415 genotype (AA or BB). Fold changes were calculated relative to the average of the CON group, with unique letter superscripts denoting statistically significant differences (*p* < 0.05).

Of the eight genes assessed in the HYP, only two were found to be differentially expressed (Figure 1). Hypothalamic expression of *solute carrier family 16 member 2* (*SLC16A2*), also known as *MCT8*, was significantly decreased in NTIS‐AA (x̃ = −1.26 fold) and NTIS‐BB (x̃ = −1.82 fold) fetuses relative to CON, and also decreased relative to the EU‐AA and EU‐BB groups (*p* < 0.01). Expression of *iodothyronine deiodinase 2* (*DIO2*) was significantly increased in EU‐BB (x̃ = 1.38 fold, *p* = 0.014) and decreased in NTIS‐AA (x̃ = −2.11 fold, *p* < 0.001) fetuses relative to CON. A third gene, *solute carrier family 16 member 10* (*SLC16A10*), also known as *MCT10*, trended upward in EU‐AA relative to NTIS‐AA and NTIS‐BB fetuses (*p* = 0.057 for both comparisons). No significant differences in hypothalamic expression of *iodothyronine deiodinase 3* (*DIO3*), *thyrotropin releasing hormone* (*TRH*), *CRH* or *thyroid hormone receptor alpha* (*THRA*) and *beta* (*THRB*) were observed among any phenotypic groups (Figure S2).

### 3.2 Pituitary Gene Expression

**FIGURE 2:**
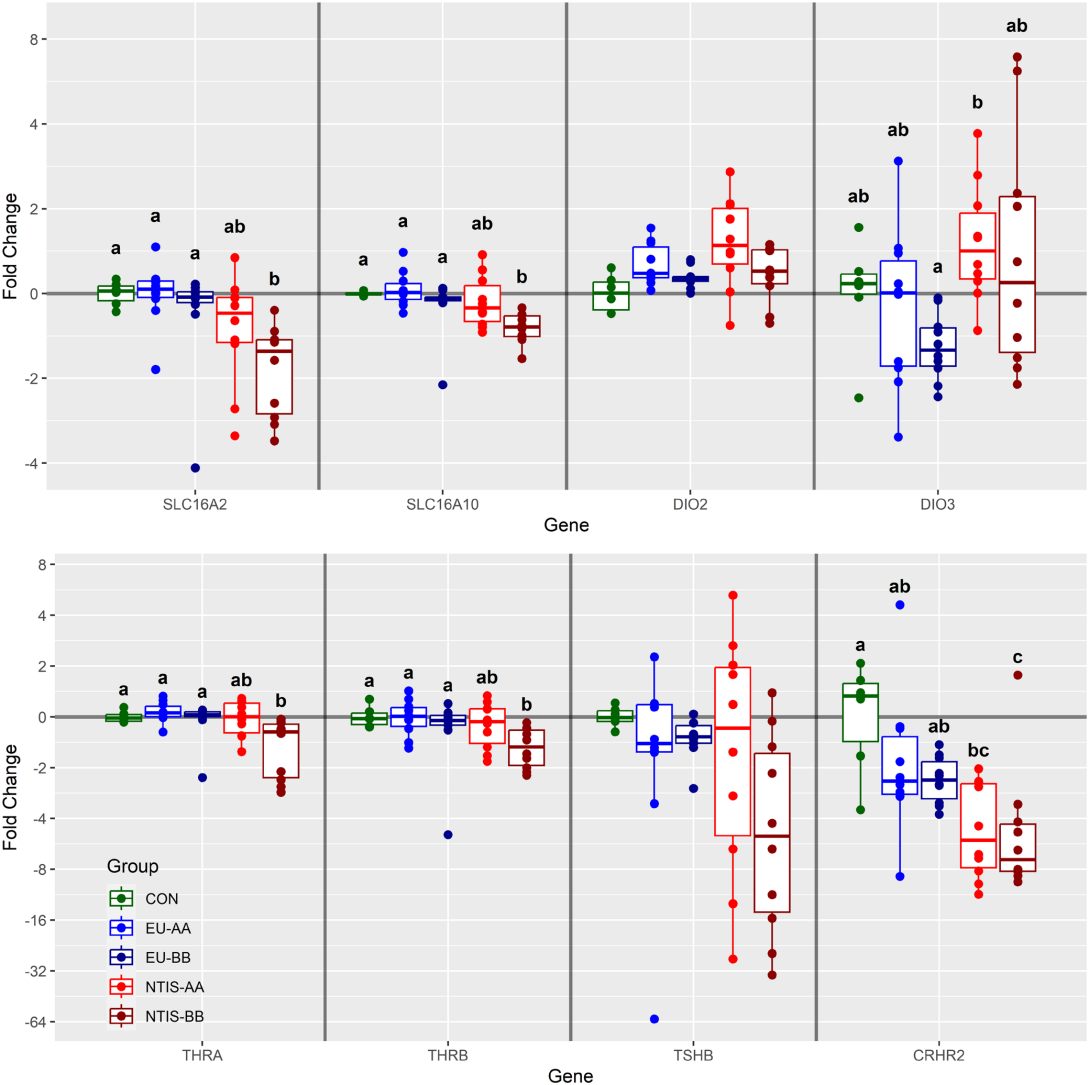
Expression of a subset of genes assessed in pituitary gland (PIT) from control (CON), euthyroid uninfected (EU), and infected low thyroid hormone (NTIS) fetuses by rs80998415 genotype (AA or BB). Fold changes were calculated relative to the average of the CON group with unique letter superscripts denoting statistically significant differences (*p* < 0.05).

Of the 11 genes examined in the PIT, six were found to be differentially expressed in at least one group (Figure 2). A significant decrease in PIT expression of both *SLC16A2* (x̃ = −1.60 fold) and *SLC16A10* (x̃ = −1.31 fold) was observed in NTIS‐BB fetuses relative to CON, EU‐AA and EU‐BB fetuses (*p* < 0.05). A similar downregulation was observed in PIT expression of *THRA* (x̃ = −1.22 fold) and *THRB* (x̃ = −1.5 fold) in NTIS‐BB fetuses relative to CON and both EU groups (*p* < 0.05). Expression of *corticotropin releasing hormone receptor 2* (*CRHR2*) was significantly decreased in both NTIS‐AA (x̃ = −5.27 fold, *p* = 0.028) and NTIS‐BB (x̃ = −6.96 fold, *p* = 0.035) groups relative to CON, with a similar trend observed in the EU‐BB group (x̃ = −2.36 fold, *p* = 0.075). Finally, a significant difference in relative expression of *DIO3* was observed between NTIS‐AA and EU‐BB fetuses (*p* = 0.004), but not between any of the other three groups. We observed no significant differences in PIT expression of the remaining five genes (Figure S3), including *DIO2*, *thyroid stimulating hormone subunit beta* (*TSHB*), *thyrotropin releasing hormone receptor* (*TRHR*), *corticotropin releasing hormone receptor 1* (*CRHR1*) and *proopiomelanocortin* (*POMC*), the latter of which encodes the prepropeptide from which ACTH is derived.

### 3.3 Thyroid Gene Expression

**FIGURE 3:**
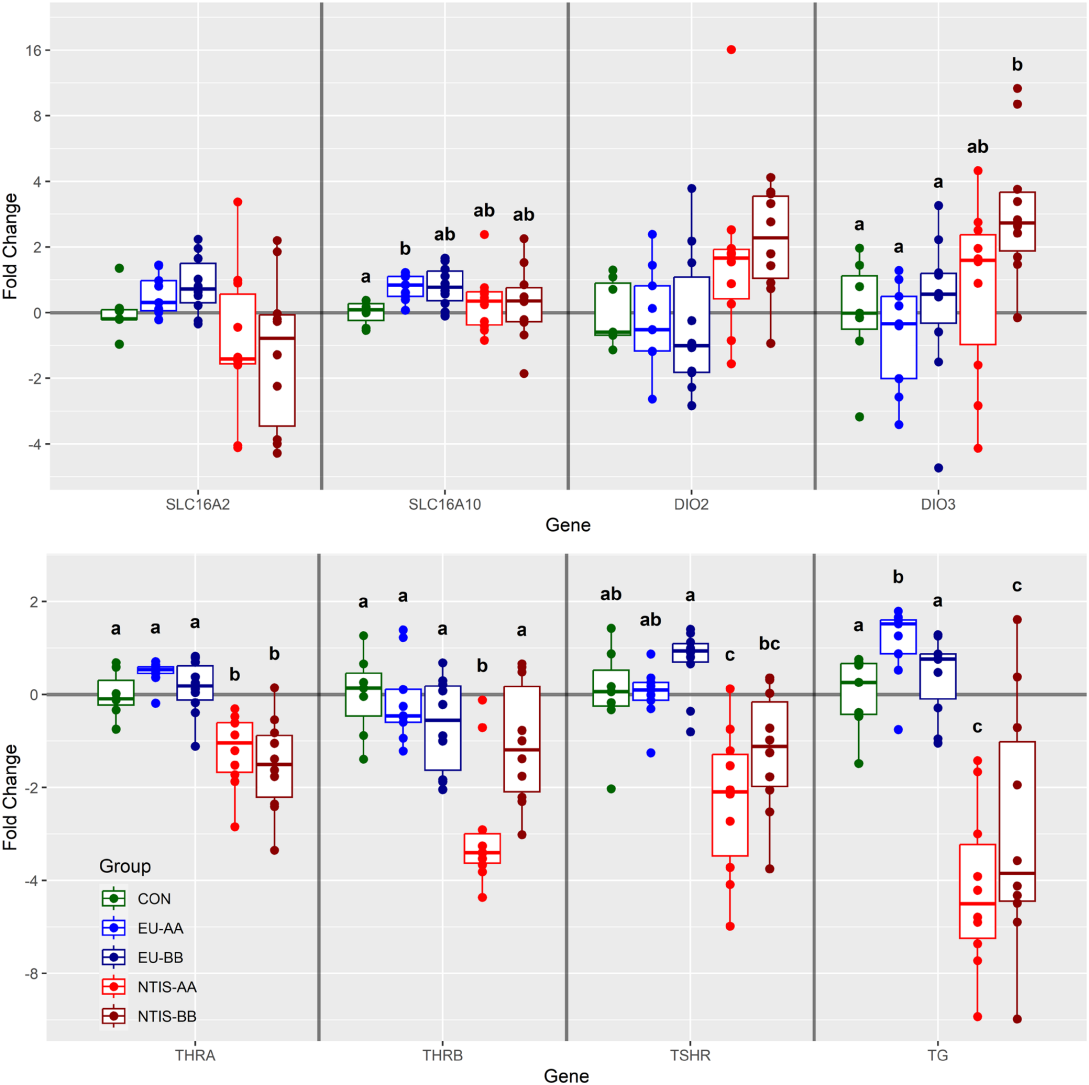
Expression of a subset of genes assessed in thyroid gland (ROID) from control (CON), euthyroid uninfected (EU), and infected low thyroid hormone (NTIS) fetuses by rs80998415 genotype (AA or BB). Fold changes were calculated relative to the average of the CON group, with unique letter superscripts denoting statistically significant differences (*p* < 0.05).

A total of nine genes were evaluated in the ROID, of which six were found to be differentially expressed in at least one fetal group (Figure 3). The most substantial dysregulation was observed for the *thyroid hormone precursor thyroglobulin* (*TG*), which was significantly upregulated in EU‐AA (x̃ = 0.59 fold, *p* = 0.033) and significantly downregulated in both NTIS‐AA (x̃ = −4.74 fold, *p* = 0.004) and NTIS‐BB (x̃ = −3.77 fold, *p* = 0.035) fetuses relative to CON. Expression of *THRA* was significantly downregulated in both NTIS‐AA (x̃ = −1.43 fold, *p* = 0.008) and NTIS‐BB (x̃ = −1.68 fold, *p* = 0.009) fetuses, while *THRB* was downregulated in NTIS‐AA (x̃ = −3.25 fold, *p* = 0.012) fetuses only when compared to all other groups. ROID expression of *thyroid stimulating hormone receptor* (*TSHR*) was also significantly downregulated in the NTIS‐AA fetuses (x̃ = −2.07 fold) relative to CON, EU‐AA and EU‐BB (*p* < 0.05) fetuses. Expression of *DIO3* was significantly upregulated in NTIS‐BB (x̃ = 2.57 fold) relative to CON, EU‐AA and EU‐BB (*p* < 0.05), and *DIO2* showed a similar trend (*p* = 0.07) toward upregulation in the NTIS‐BB group (x̃ = 2.23 fold). *SLC16A10* showed a marginal (x̃ = 1.34 fold), but significant (*p* = 0.043), upregulation in EU‐AA relative to the CON group alone. Expression of the two remaining genes assessed in the ROID, *iodothyronine deiodinase 1* (*DIO1*) (Figure S4) and *SLC16A2*, was not significantly different among any of the phenotypic groups.

### 3.4 Adrenal Gene Expression

**FIGURE 4:**
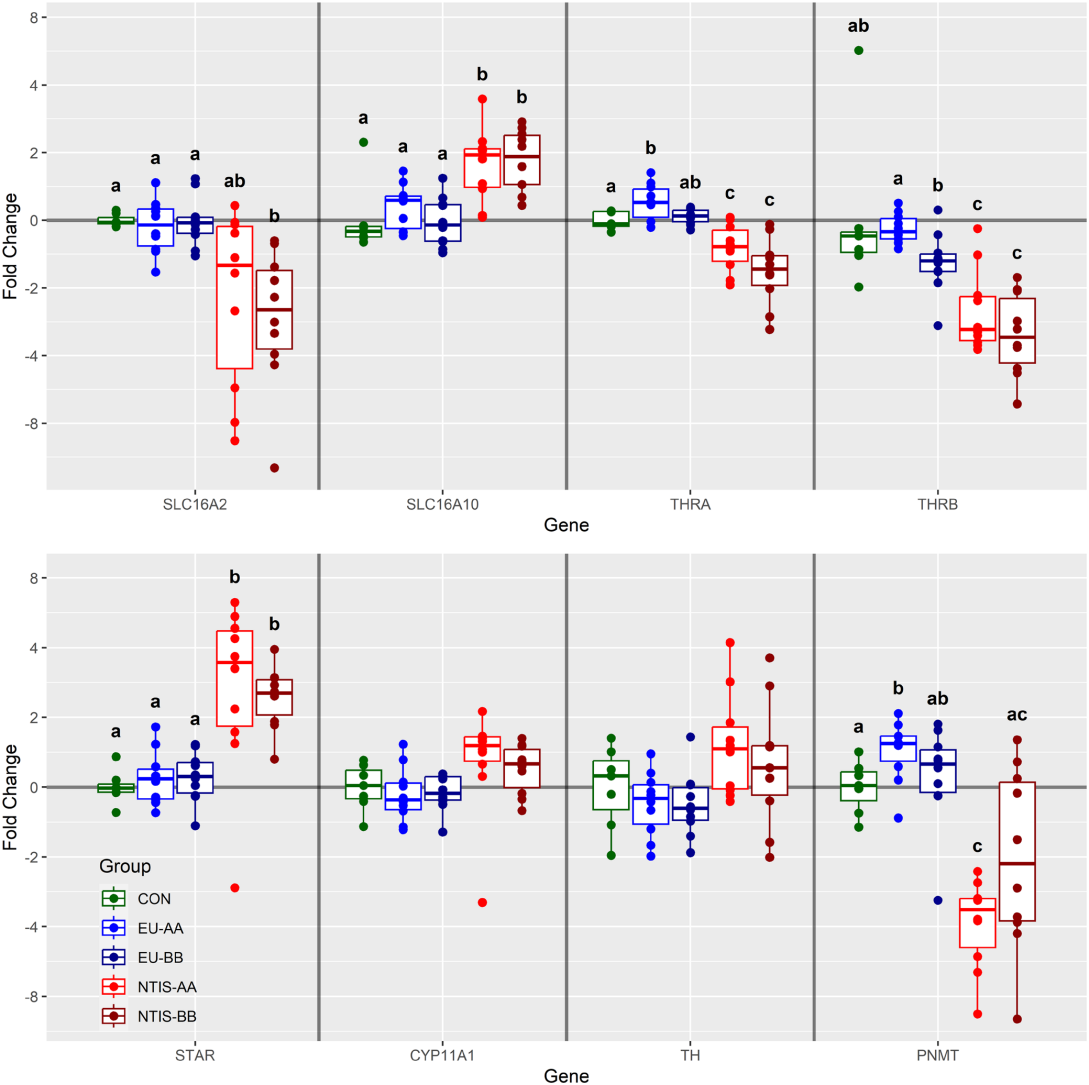
Expression of a subset of genes assessed in adrenal glands (ADR) from control (CON), euthyroid uninfected (EU), and infected low thyroid hormone (NTIS) fetuses by rs80998415 genotype (AA or BB). Fold changes were calculated relative to the average of the CON group, with unique letter superscripts denoting statistically significant differences (*p* < 0.05).

A total of 14 genes were evaluated in the ADR, six of which were found to be differentially expressed (Figure 4). *SLC16A2* was significantly downregulated in NTIS‐BB fetuses (x̃ = −2.47 fold) relative to CON, EU‐AA and EU‐BB fetuses (*p* < 0.01), with a similar trend noted for the NTIS‐AA group (x̃ = −1.58 fold *p* = 0.09). In contrast, *SLC16A10* was significantly upregulated in both NTIS‐AA (x̃ = −1.93 fold *p* = 0.019) and NTIS‐BB (x̃ = −1.95 fold *p* = 0.014) fetuses relative to CON, EU‐AA and EU‐BB fetuses. Expression of *THRA* was significantly downregulated in both NTIS‐AA (x̃ = −1.31 fold, *p* = 0.021) and NTIS‐BB (x̃ = −1.65 fold, *p* = 0.006) fetuses relative to CON and EU‐BB, with a marginal but significant upregulation in EU‐AA (x̃ = 1.20 fold, *p* = 0.040) fetuses relative to CON only. *THRB* was similarly downregulated in NTIS‐AA (x̃ = −3.06 fold) and NTIS‐BB (x̃ = −3.30 fold) fetuses relative to CON, EU‐AA and EU‐BB (*p* < 0.01), while EU‐BB fetuses showed a significant downregulation (x̃ = −1.5 fold, *p* = 0.012) only relative to EU‐AA. Expression of the gene encoding the *steroidogenic acute regulatory protein* (*STAR*) was significantly upregulated in both NTIS‐AA (x̃ = 3.46 fold, *p* < 0.01) and NTIS‐BB (x̃ = 2.55 fold, *p* < 0.05) fetuses relative to CON, EU‐AA and EU‐BB fetuses. *Cytochrome P450 family 11 subfamily A member 1* (*CYP11A1*), also part of the steroidogenic pathway, showed a trending upregulation in NTIS‐AA (x̃ = 1.51 fold) fetuses relative to the CON (*p* = 0.073) and EU‐AA and EU‐BB (*p* = 0.057) groups. Expression of *phenylethanolamine N‐methyltransferase* (*PNMT*) was significantly downregulated in NTIS‐AA fetuses (x̃ = −3.36 fold) relative to CON, EU‐AA and EU‐BB (*p* < 0.01), with an additional downregulation in the NTIS‐BB group (x̃ = −2.08 fold, *p* = 0.015) relative to EU‐AA only. A trend toward minor upregulation in *tyrosine hydroxylase* (*TH*) expression was observed in NTIS‐AA fetuses relative to the EU‐AA and EU‐BB groups (*p* = 0.057). Expression of the remaining six genes assessed in the ADR (Figure S5), including *DIO2*, *DIO3*, *dopamine beta‐hydroxylase* (*DBH*), *dopa decarboxylase* (*DDC*), *cytochrome P450 family 11 subfamily B member* (*CYP11B*), and *melanocortin 2 receptor* (*MC2R*), was not significantly different among any phenotypic groups.

### 3.5 Pathway Analysis of the Hypothalamic–Pituitary‐Thyroid/−Adrenal Axes

**FIGURE 5:**
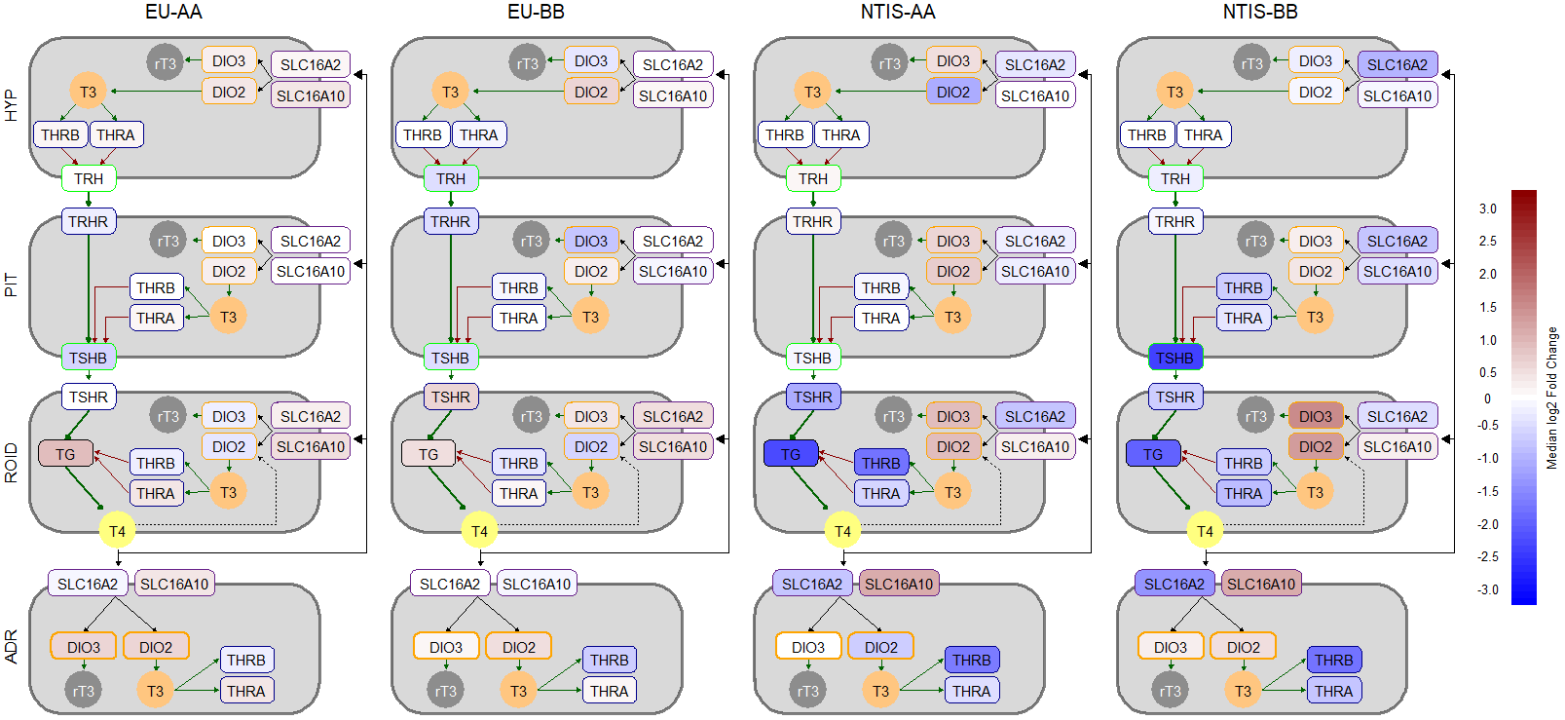
Multitissue heat map of gene expression results visualizing alterations in thyroid hormone regulation, signaling, and metabolism across the hypothalamus (HYP), pituitary (PIT), thyroid (ROID), and adrenals (ADR) from euthyroid uninfected (EU) and infected low thyroid hormone (NTIS) fetuses by rs80998415 genotype (AA or BB). Data within each box (labeled by gene) represents the median log2 fold change of gene expression relative to control fetuses, with box fill color indicating the direction and scale of each dysregulation.

**FIGURE 6:**
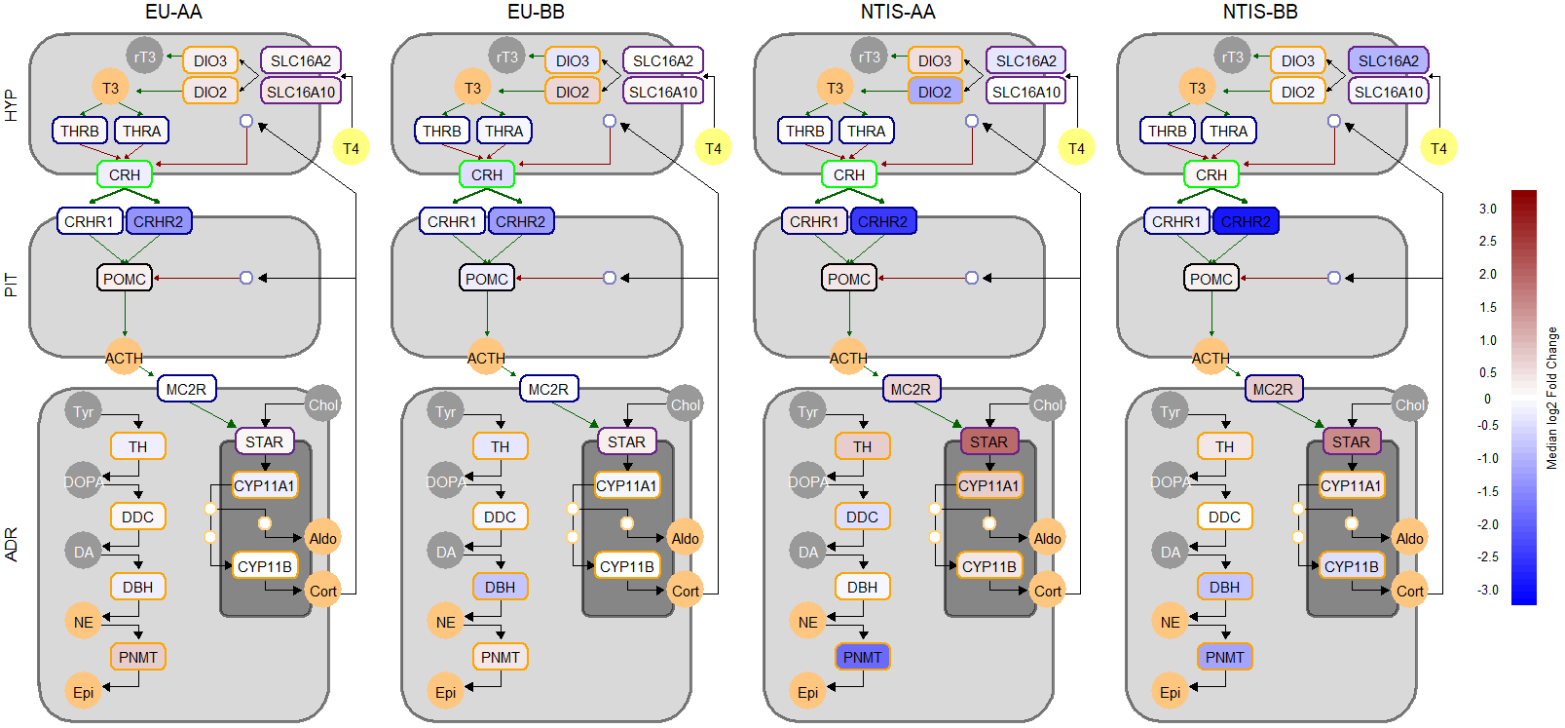
Multitissue heat map of gene expression results visualizing alterations in regulation, signaling and hormone synthesis across the hypothalamus (HYP), pituitary (PIT), and adrenals (ADR) from euthyroid uninfected (EU) and infected low thyroid hormone (NTIS) fetuses by rs80998415 genotype (AA or BB). Data within each box (labeled by gene) represents the median log2 fold change of gene expression relative to control fetuses, with box fill color indicating the direction and scale of each dysregulation. Abbreviations: Adrenocorticotropic hormone (ACTH), tyrosine (Tyr), dihydroxyphenylalanine (DOPA), dopamine (DA), norepinephrine (NE), epinephrine (Epi), cholesterol (Chol), aldosterone (Aldo), cortisol (Cort).

To better understand the collective impact of gene expression changes on thyroid hormone regulation within the HPTA axis, a heat map was constructed (Figure 5) to visualize the gene expression results across multiple tissues. The results indicate that fetal infection and pathogen‐induced suppression in circulating thyroid hormone levels is associated with widespread alterations in thyroid hormone bioavailability and bioactivity, as indicated by changes in expression of key thyroid hormone transporters, receptors, and deiodinases. Additionally, alterations within the ROID, including decreased expression of *TG* and *TSHR*, indicate a decreased productive capacity for thyroid hormones, with the overall scale of this dysregulation modulated by the rs80998415 genotype. To get an equivalently broad perspective into the functional impact of infection and reduced thyroid hormone availability on ADR activity, a similar multitissue heat map of gene expression was constructed for the canonical HPA axis (Figure 6). Overall, the pathway suggests a limited impact of phenotypic group on the central regulatory mechanisms, but evidence of steroidogenic upregulation as well as modulation of the catecholamine pathway in response to infection is evident. As with the HPTA axis, the degree of dysregulation within the canonical HPA axis is modulated by the rs80998415 genotype.

### 3.6 Serum Endocrine Effects

**FIGURE 7:**
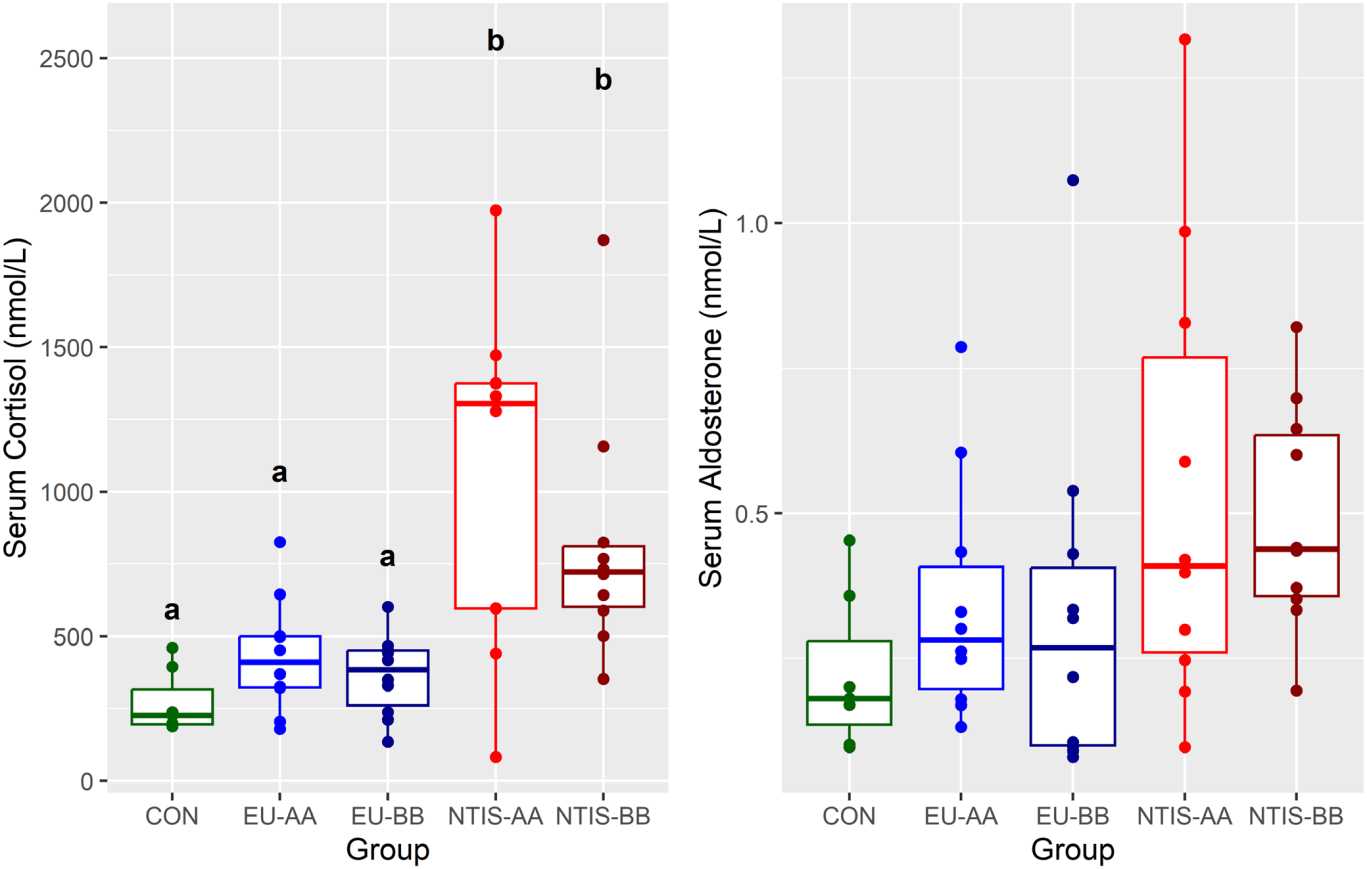
Serum cortisol (A) and aldosterone in control (CON), euthyroid uninfected (EU), and infected low thyroid hormone (NTIS) fetuses by rs80998415 genotype (AA or BB) with unique letter superscripts denoting statistically significant differences (*p* < 0.05).

By design of the fetal selection, serum T3 and T4 levels were not significantly different between CON and fetuses selected for either euthyroid group (EU‐AA & EU‐BB). In contrast, both T3 and T4 were significantly reduced in both NTIS groups (NTIS‐AA & NTIS‐BB) relative to the CON and EU groups (Table 1). To understand how observed changes within the HPA axis might impact concentrations of circulating endocrine signals, we next measured fetal serum cortisol and aldosterone levels (Figure 7). Cortisol in EU‐AA (x̃ = 410.78 nmol/L) and EU‐BB (x̃ = 383.97 nmol/L) fetuses did not significantly differ from CON (x̃ = 226.43 nmol/L), but cortisol levels were significantly (*p* < 0.05) elevated in both NTIS‐AA (x̃ = 1304.81 nmol/L) and NTIS‐BB fetuses (x̃ = 723.35 nmol/L) relative to the CON, EU‐AA and EU‐BB groups. There was no significant difference seen in serum aldosterone levels between CON (x̃ = 0.18 nmol/L), EU‐AA (x̃ = 0.28 nmol/L), EU‐BB (x̃ = 0.27 nmol/L), NTIS‐AA (x̃ = 0.41 nmol/L) or NTIS‐BB (x̃ = 0.44 nmol/L) fetuses.

## 4 Discussion

In utero development involves a coordinated series of ontological events and is highly susceptible to perturbation by chemical and pathological teratogens. To prevent such disruption, the conceptus is shielded from external factors by a normally restrictive placenta; however, a number of teratogens, including viral pathogens, are able to circumvent this barrier. During late gestation, PRRSV is capable of vertical transmission across the epitheliochorial placenta of the pig, resulting in a productive infection in a subset of the fetal population. Viral strains which typically cause limited clinical signs in the dam create critical illness in the infected fetuses (Ladinig et al. 2015), resulting in developmental disruption and fetal compromise. Much like other members of the Nidovirales order which have been shown to induce NTIS following postnatal infection (Zhang et al. 2021), fetal PRRSV infection causes a profound reduction in circulating thyroid hormone levels (Pasternak et al. 2020b). This reduction in hormone availability is associated with decompensatory peripheral metabolism of thyroid hormone (Ison et al. 2022), a common characteristic of NTIS (Langouche et al. 2019). Of particular interest, the degree of circulating T3 suppression in resilient PRRSV infected fetuses is significantly greater than their equally infected but susceptible counterparts (Pasternak et al. 2020b). However, the underlying alterations to central regulatory mechanisms controlling hormone availability in this condition have not been previously described.

A previous genome‐wide association study (GWAS) performed following the largest controlled reproductive PRRSV challenge conducted to date identified rs80998415 as a SNP significantly associated with fetal response to PRRSV infection (Yang et al. 2016). This intergenic SNP, originally referred to as DRGA0008048, was estimated to explain 34.6% of the genetic variance in fetal viability (Yang et al. 2016). Subsequent investigation of this SNP found it located on *Sus scrofa* chromosome 7 (SSC7) roughly 15 kb from *DIO2* and 700 kb from *TSHR*. With no logical biological association identified for other nearby genes such as *centrosomal protein 128* (*SEP128*) and *neurexin 3* (*NRXN3*), thyroid hormone regulation was identified as a likely causative pathway. Targeted resequencing of *DIO2* and *TSHR* genes has identified a number of polymorphisms in high linkage disequilibrium with the rs80998415 SNP (Ko et al. 2024). The functional association between this SNP and the NTIS‐like response observed in PRRSV infected fetuses was further strengthened by a targeted post GWAS experiment which found an association between rs80998415 and circulating fetal T4 levels (Ko et al. 2022), warranting further investigation into the influence of this SNP on stress response within the HPTA axis.

### 4.1 Impact of Fetal PRRSV Infection on the Hypothalamic–Pituitary‐Thyroid Axis

In the present study, the most substantial change to central thyroid hormone synthesis in the HPT axis occurred within the ROID where both *TSHR* and *TG* were downregulated in NTIS fetuses. Thyroid hormones are synthesized from the tyrosine residues in the TG protein, which are iodinated and conjugated within the eosinophilic colloid and then liberated from the protein backbone within the follicular epithelial cells (Koibuchi 2012). Tissue‐specific expression of the *TG* gene is regulated by the TTF1 transcription factor which is produced in response to TSHR stimulation and acts on a pair of binding sites within the *TG* promoter (Civitareale et al. 1989). Interestingly, TTF1 has also been shown to negatively regulate expression of *TSHR* in response to the ligand (Shimura et al. 1994). Thus, the combined downregulation of both *TG* and *TSHR* observed within the NTIS fetuses in the present study is unlikely to result from decreased TSH signaling and would instead suggest a local effect within the ROID.

Along similar lines, the present results suggest an allostatic response of thyroid hormone metabolism to fetal infection, whereby the observed transcriptional changes are a likely cause of, and not a result of, the decrease in thyroid hormone levels observed in NTIS fetuses. In contrast to prior studies in drug‐induced models of porcine fetal hypothyroidism (Ison et al. 2023), which show compensatory responses to thyroid hormone metabolism, NTIS is associated with decompensatory thyroid hormone metabolism and an alteration in the normal metabolic setpoint of the HPT axis (Chatzitomaris et al. 2017). In the present study, fetal infection was associated with increased expression of *DIO3* in the ROID of NTIS‐BB fetuses, and decreased expression of *DIO2* in the HYP of NTIS‐AA fetuses, which may contribute to increased rT3 levels and decreased conversion of T4 to T3, respectively. This, along with the widespread downregulations observed in thyroid hormone receptor expression, would contribute to a reduction in bioactive thyroid hormone signaling and metabolic activity, consistent with NTIS.

While the exact mechanism mediating these transcriptional changes is unclear, one potential contributor may be the robust immune response exhibited in PRRSV‐infected fetuses, which includes both a local immune response in infected tissues, as well as a systemic immune response (Pasternak et al. 2020a; Rowland 2010; Rudy et al. 2024). In highly infected fetuses, serum inflammatory signals including IFNα, IFNγ, TNFα, and CCL2 are significantly elevated, with additional upregulation observed in the expression of transcripts for *IFNB*, *CCL5*, *CXCL10*, and *IL10* in both spleen and thymic tissue (Pasternak et al. 2020a). Similar inflammatory responses are also observed in nonlymphoid organs including the heart, liver, and kidney (Rudy et al. 2024; Walker et al. 2024) and the fetal portion of the placenta (Van Goor et al. 2020). Immune activation has been shown to be a major contributor to NTIS, with one study showing that NTIS can be induced in the pig via intravenous administration of lipopolysaccharide (Castro et al. 2013), and previous studies identifying cytokines such as IL6 to be closely associated with an NTIS‐like state (Abo‐Zenah et al. 2008). Further, NTIS has also been frequently reported in cases of heart failure and is associated with adverse outcomes (Qi et al. 2023). While the causative mechanism behind type 1 allostasis has not been established, it appears to involve multilevel effects within the HPT axis and other endocrine pathways (Čulić 2024), which is consistent with the present observations in the fetal pig. While evidence of overt heart failure in the PRRSV infected fetal pig has not been reported, there is a growing body of transcriptional evidence indicating altered cardiac physiology in infected fetuses (Malgarin et al. 2021; Mulligan et al. 2022; Pasternak et al. 2020b; Walker et al. 2024), which may be of interest for further studies.

### 4.2 Impact of Fetal PRRSV Infection on the Hypothalamic–Pituitary–Adrenal Axis

Similar to the locality of dysregulation occurring in the ROID in the HPT axis, the ADR appeared to be the most impacted organ in the HPA axis. The most marked dysregulation observed in the steroidogenesis pathway was *STAR*, which was upregulated in both NTIS groups. As STAR regulates the rate‐limiting step of steroidogenesis (Manna et al. 2009), this upregulation likely represents increased availability of cholesterol for conversion into steroid hormones, which may have contributed to the increase in circulating cortisol concomitantly observed in both NTIS groups. As stated previously, vertical transmission of PRRSV to the developing conceptus is associated with a complex physiological response, including immune activation (Pasternak et al. 2020a; Rowland 2010). One prior report in humans indicates that cytokines, namely IL2, may drive increased cortisol secretion (Roelfsema et al. 2020), which may have contributed to the observed increase in cortisol in NTIS fetuses. Similarly, meconium staining, which is frequently observed in fetuses with high PRRS viral load, has been associated with fetal distress and elevated umbilical cortisol during parturition (Rabiepoor et al. 2019). Thus, the present experiment cannot distinguish between a direct effect of viral infection on the HPA axis and associated cortisol secretion, or an indirect response mediated by inflammatory signaling or endocrinological stress response. Regardless, the observed changes in cortisol levels in NTIS fetuses are consistent with prior reports in high viral load PRRSV‐infected fetuses (Ko et al. 2024). As previously hypothesized, and of practical importance to the swine industry, these increased cortisol levels may contribute to fetal compromise and/or premature birth in infected litters.

In addition to their role in steroidogenesis, the ADR act as a major site for production and secretion of catecholamines, a pathway that begins with the precursor tyrosine and is regulated by endogenously expressed genes including *TH*, *DDC*, *DBH*, and *PNMT*. In the current study, we saw modulations in catecholamine production as a result of infection, with downregulations of ADR *PNMT* expression seen in NTIS fetuses suggesting increased accumulation of norepinephrine and decreased production of epinephrine. Similar effects on catecholamine production have previously been observed in cases of fetal hypoxia (Mamet et al. 2002), and may also result from increased levels of glucocorticoids (Adams et al. 1999), the latter of which is consistent with the increase in steroidogenesis also observed in the NTIS groups in the present study. Late gestation PRRSV challenge has been associated with histopathological lesions within the maternal fetal interface (Novakovic et al. 2016), which may result in systemic fetal hypoxia. In addition, there is at least some evidence of cardiovascular hypoxia within PRRSV infected fetuses (Malgarin et al. 2021). Further, there is prior evidence of a bidirectional regulatory relationship between catecholamines and the renin‐angiotensin‐aldosterone system (RAAS), including stimulatory effects of epinephrine and norepinephrine on renin (Vandongen and Greenwood 1975) and aldosterone (Horiuchi et al. 1987) secretion, and induction of catecholamine release as a result of angiotensin II activity (Powis and O'Brien 1991). Previously, we reported an increase in cardiovascular expression of *angiotensinogen*, the precursor to all bioactive RAAS peptides, as a result of fetal PRRSV infection (Pasternak et al. 2020b). This suggests that fetal infection may increase local cardiac RAAS activity and angiotensin II production, which may lead to adverse cardiovascular effects such as hypertrophy and hypertension (Crowley et al. 2006; Matsuda et al. 2015).

### 4.3 Interactions Between the HPT and HPA Axes

While many of the observed effects for the HPT and HPA axes were localized largely to the ROID and ADR, respectively, dysregulations in expression of the solute carrier genes were observed across all four studied tissues. The two assessed solute carriers, *SLC16A2* and *SLC16A10*, are responsible for both cellular intake and efflux of thyroid hormones and are more commonly referred to as *MCT8* and *MCT10*, respectively (van der Deure et al. 2010). As *SLC16A2* has a high specificity for thyroid hormones, mutations in this gene are known to lead to a wide array of congenital detriments including mental deficits and abnormal circulating thyroid hormone levels (Müller and Heuer 2012). Consistent with our prior findings in the PRRSV‐infected fetal heart (Pasternak et al. 2020b), we found fetal infection to be associated with decreased HYP, PIT, and ADR expression of *SLC16A2*. Interestingly, the present study further revealed that these changes were dependent on fetal genotype, with expression significantly decreased across all three of these tissues in the NTIS‐BB group, and only significantly decreased in the HYP of the NTIS‐AA group. Similarly, while *SLC16A10* expression was uniquely upregulated in the ADR of both NTIS groups, only NTIS‐BB fetuses exhibited decreases in *SLC16A10* expression in the PIT, suggesting an effect of fetal genotype in the response to infection. The role of these two solute carrier genes, as well as the potential interaction between them, has previously been shown to be tissue‐specific. For example, concomitant deficiencies in both *SLC16A2* and *SLC16A10* are able to partially ameliorate the systemic phenotype of *SLC16A2*‐alone knockout mice, while exacerbating effects in certain tissues such as the liver and kidneys (Müller et al. 2014). As such, more research is needed to fully understand the impact of the observed transcriptional effects, although one could hypothesize that the impaired thyroid hormone transport resulting from alterations in solute carrier expression may disrupt the crosstalk between tissues within the HPTA axis.

In conclusion, our results show that fetal PRRSV infection is associated with altered transcriptional activity within both the HPT and HPA axes. Consistent with NTIS, we observed evidence of decreased tissue sensitivity to thyroid hormones in infected fetuses, with dysregulations in the expression of key thyroid hormone receptor and deiodinase genes. We additionally observed evidence of reduced thyroid hormone productive capacity within the ROID itself, with downregulations in the expression of both *TSHR* and *TG*. In the HPA axis, fetal infection resulted in apparent dysregulations in key pathways regulating both catecholamine production and steroidogenesis, with the latter further evidenced by increased circulating cortisol levels in NTIS fetuses. Of practical importance, the severity of the observed effects was partially dependent on the rs80998415 genotype. Collectively, these results not only provide support for the critical role of the rs80998415 SNP in regulating the response of key endocrine systems to fetal PRRSV infection, but also help characterize the central and peripheral mechanisms regulating the NTIS‐like state observed in fetal PRRSV infection.

## Author Contributions

J.A.P., G.S.P., and J.C.S.H. conceived the original animal study and underlying hypothesis. G.H. selected fetal populations and conducted the laboratory analysis. J.A.P. and A.A.S. conducted the statistical analysis, developed the multitissue heat mapping approach, and drafted the manuscript, which was reviewed by G.H., G.S.P., and J.C.S.H.

## Funding

Funding for the animal and molecular work was provided by Genome Alberta through the A3GP program (Project ALGP47) with in‐kind contributions and support from Fast Genetics. Data analysis and dissemination was supported by the Foundational and Applied Science Program, project award nos. 2023‐67015‐39338 and 2023‐67015‐39080 from the U.S. Department of Agriculture's National Institute of Food and Agriculture.

## Conflicts of Interest

The authors declare no conflicts of interest.

## Supporting information


**Figure S1:** Images of H&E stained histological sections demonstrating the specific isolation of fetal diencephalon (A) including hypothalamus (*), pituitary (B) including posterior neurohypophysis (Ŧ) and anterior adenohypophysis (¤), adrenal (C) with intact cortex (ф) surrounding the medulla (§), and the thyroid (D) with characteristic follicles filled with eosinophilic colloid (δ).

**Figure S2:** Expression of additional genes assessed in the diencephalon including the hypothalamus (HYP) from control (CON), euthyroid uninfected (EU) and infected low thyroid hormone (NTIS) fetuses by rs80998415 genotype (AA or BB). Fold changes were calculated relative to the average of the CON group, and no significant differences in expression observed between groups.

**Figure S3:** Expression of additional genes assessed in the pituitary gland (PIT) from control (CON), euthyroid uninfected (EU) and infected low thyroid hormone (NTIS) fetuses by rs80998415 genotype (AA or BB).

**Figure S4:** Expression of *iodothyronine deiodinase 1* (*DIO1*) in the thyroid gland (ROID) from control (CON), euthyroid uninfected (EU) and infected low thyroid hormone (NTIS) fetuses by rs80998415 genotype (AA or BB).

**Figure S5:** Expression of additional genes assessed in the adrenal gland (ADR) from control (CON), euthyroid uninfected (EU) and infected low thyroid hormone (NTIS) fetuses by rs80998415 genotype (AA or BB).

## Data Availability

The data supporting the findings of this study are available from the corresponding author upon reasonable request.
