# Supplementary material for: Hypothalamic–Pituitary–Thyroid and Adrenal Axis Modulation in Response to Fetal Porcine Reproductive and Respiratory Virus Infection
Source: Compr Physiol. 2026 Feb 9;16(1):e70112. doi: 10.1002/cph4.70112 (PMC12886161; doi:10.1002/cph4.70112)
Supplement: Supplementary file 1 — Figure S1: Images of H&E stained histological sections demonstrating the specific isolation of fetal diencephalon (A) including hypothalamus (*), pituitary (B) including posterior neurohypophysis (Ŧ) and anterior adenohypophysis (¤), adrenal (C) with intact cortex (ф) surrounding the medulla (§), and the thyroid (D) with characteristic follicles filled with eosinophilic colloid (δ). [file CPH4-16-e70112-s001.docx]

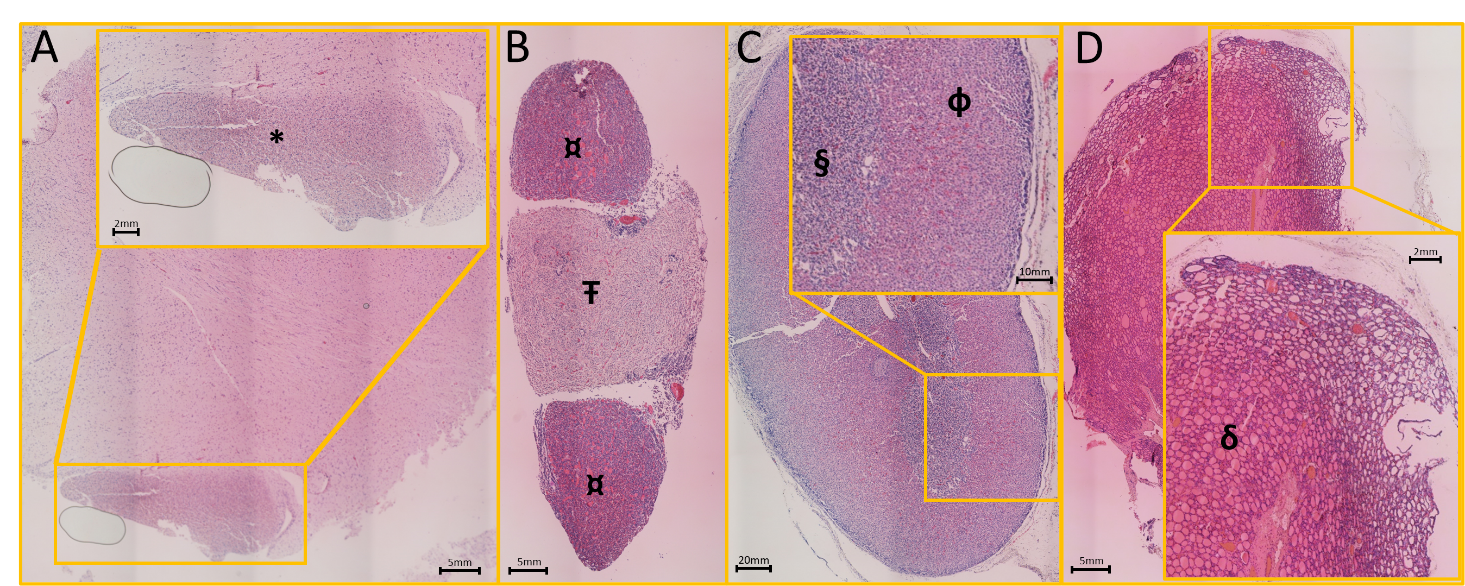


Supplemental Figure 1: Images of H&E stained histological section demonstrating the specific isolation of fetal diencephalon (A) including hypothalamus (*), pituitary (B) including posterior neurohypophysis (Ŧ) and anterior adenohypophysis (¤), adrenal (C) with intact cortex(ф) surrounding the medulla (§), and the thyroid (D) with characteristic follicles filled with eosinophilic colloid (δ).
