# Supplementary figures and images for: Hypothalamic–Pituitary–Thyroid and Adrenal Axis Modulation in Response to Fetal Porcine Reproductive and Respiratory Virus Infection
Source: Compr Physiol. 2026 Feb 9;16(1):e70112. doi: 10.1002/cph4.70112 (PMC12886161; doi:10.1002/cph4.70112)

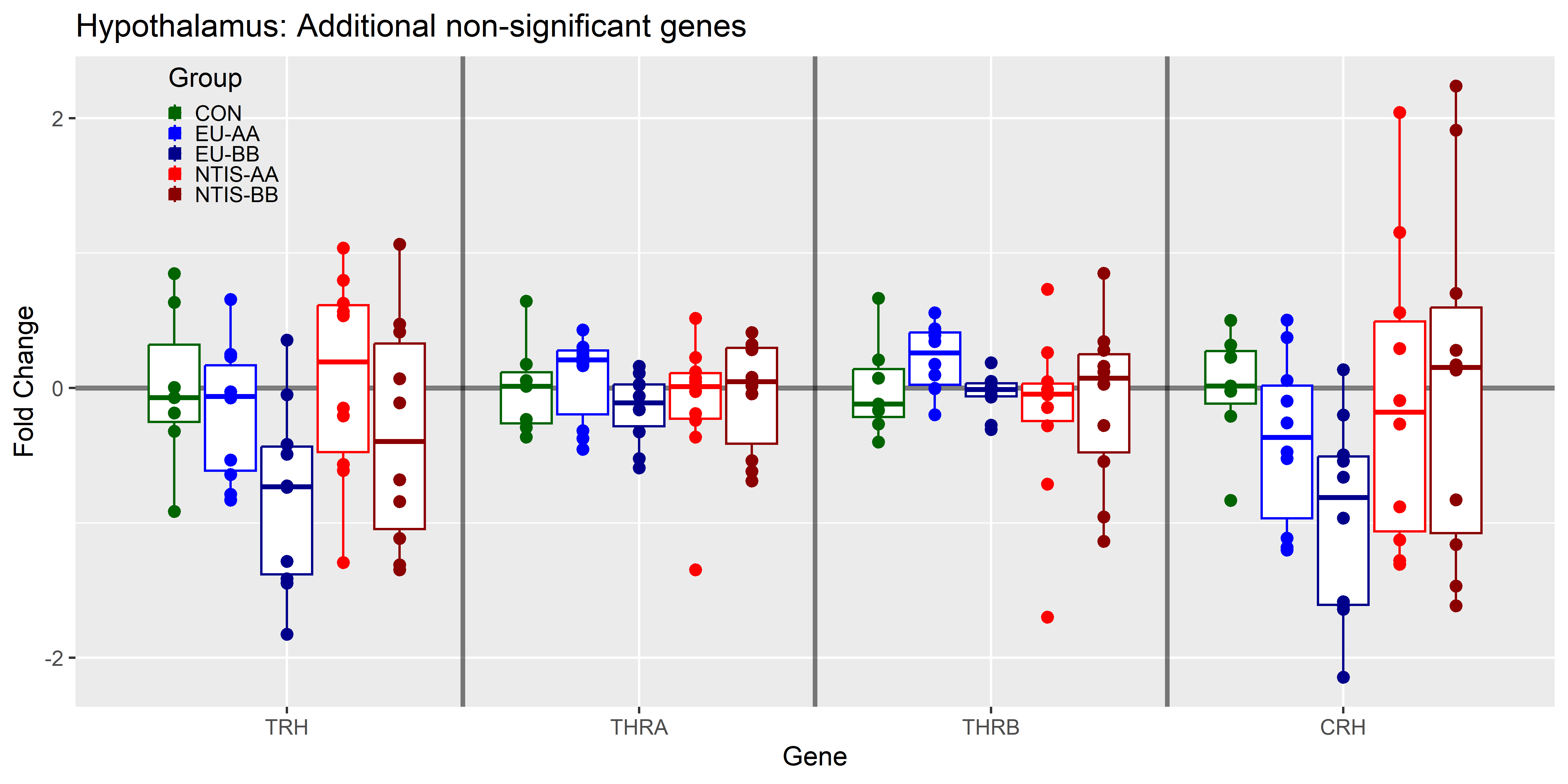

Supplement: Supplementary file 2 — Figure S2: Expression of additional genes assessed in the diencephalon including the hypothalamus (HYP) from control (CON), euthyroid uninfected (EU) and infected low thyroid hormone (NTIS) fetuses by rs80998415 genotype (AA or BB). Fold changes were calculated relative to the average of the CON group, and no significant differences in expression observed between groups. [file CPH4-16-e70112-s004.tiff]

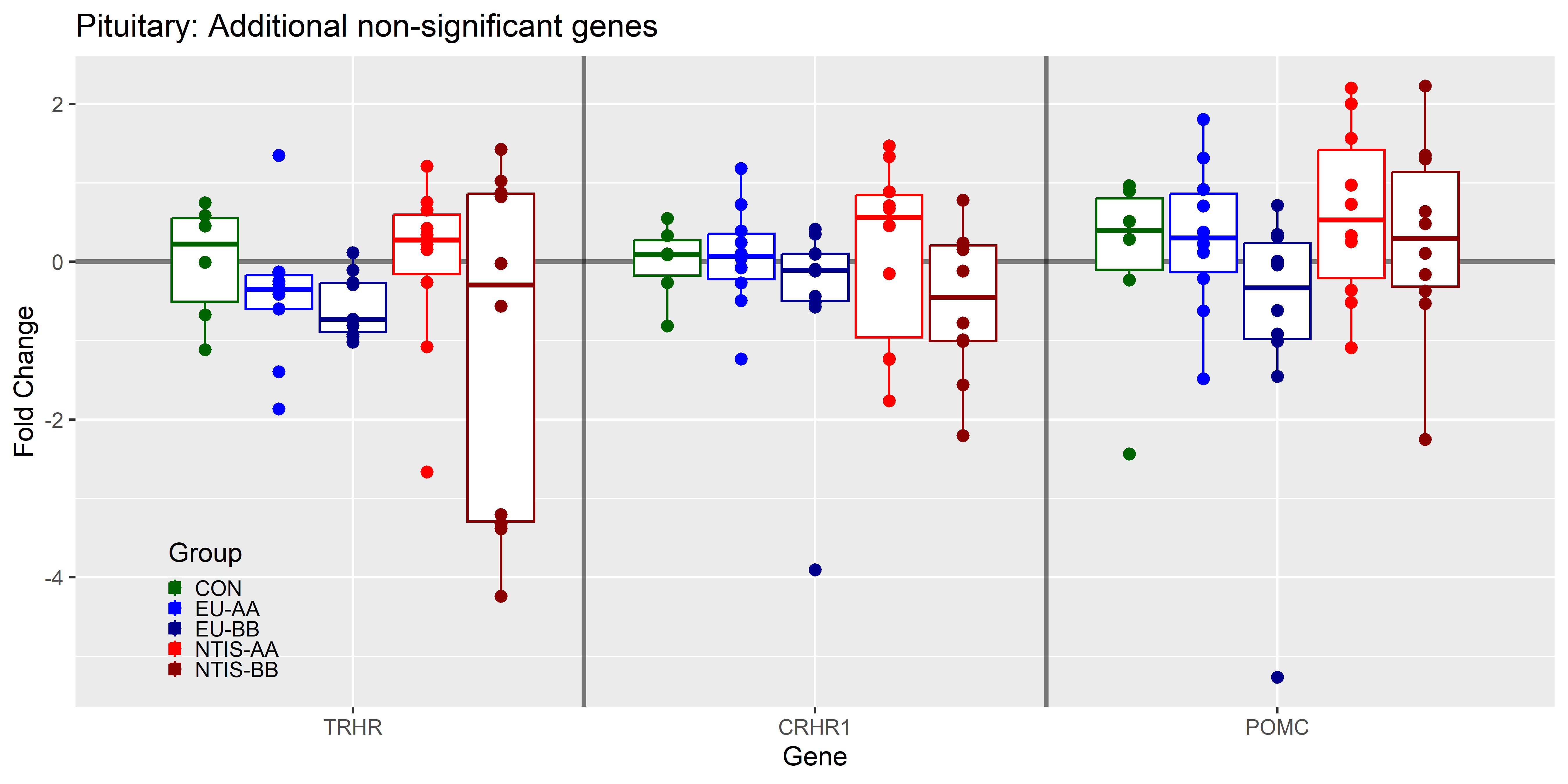

Supplement: Supplementary file 3 — Figure S3: Expression of additional genes assessed in the pituitary gland (PIT) from control (CON), euthyroid uninfected (EU) and infected low thyroid hormone (NTIS) fetuses by rs80998415 genotype (AA or BB). [file CPH4-16-e70112-s005.tiff]

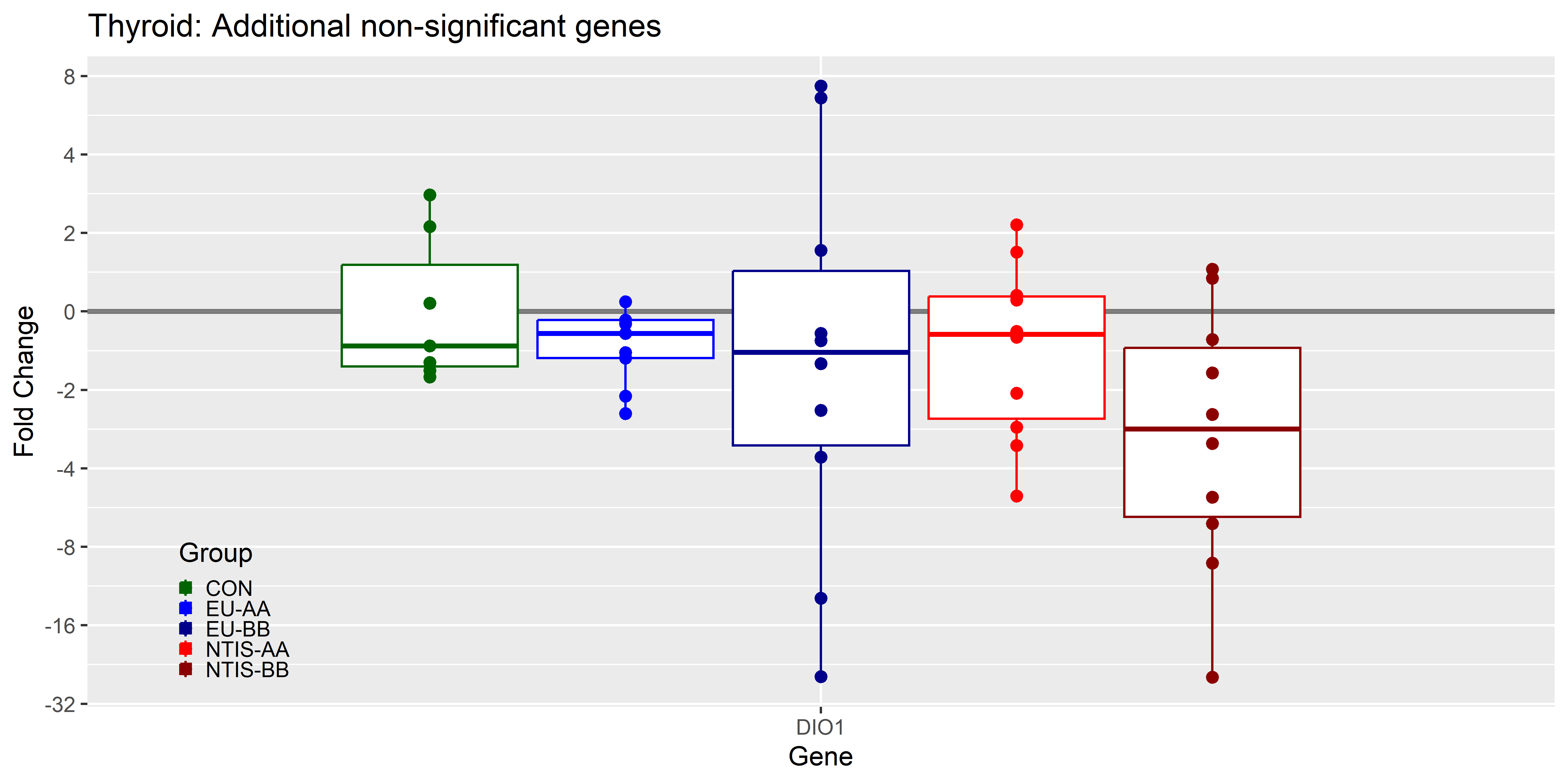

Supplement: Supplementary file 4 — Figure S4: Expression of iodothyronine deiodinase 1 (DIO1) in the thyroid gland (ROID) from control (CON), euthyroid uninfected (EU) and infected low thyroid hormone (NTIS) fetuses by rs80998415 genotype (AA or BB). [file CPH4-16-e70112-s002.tiff]

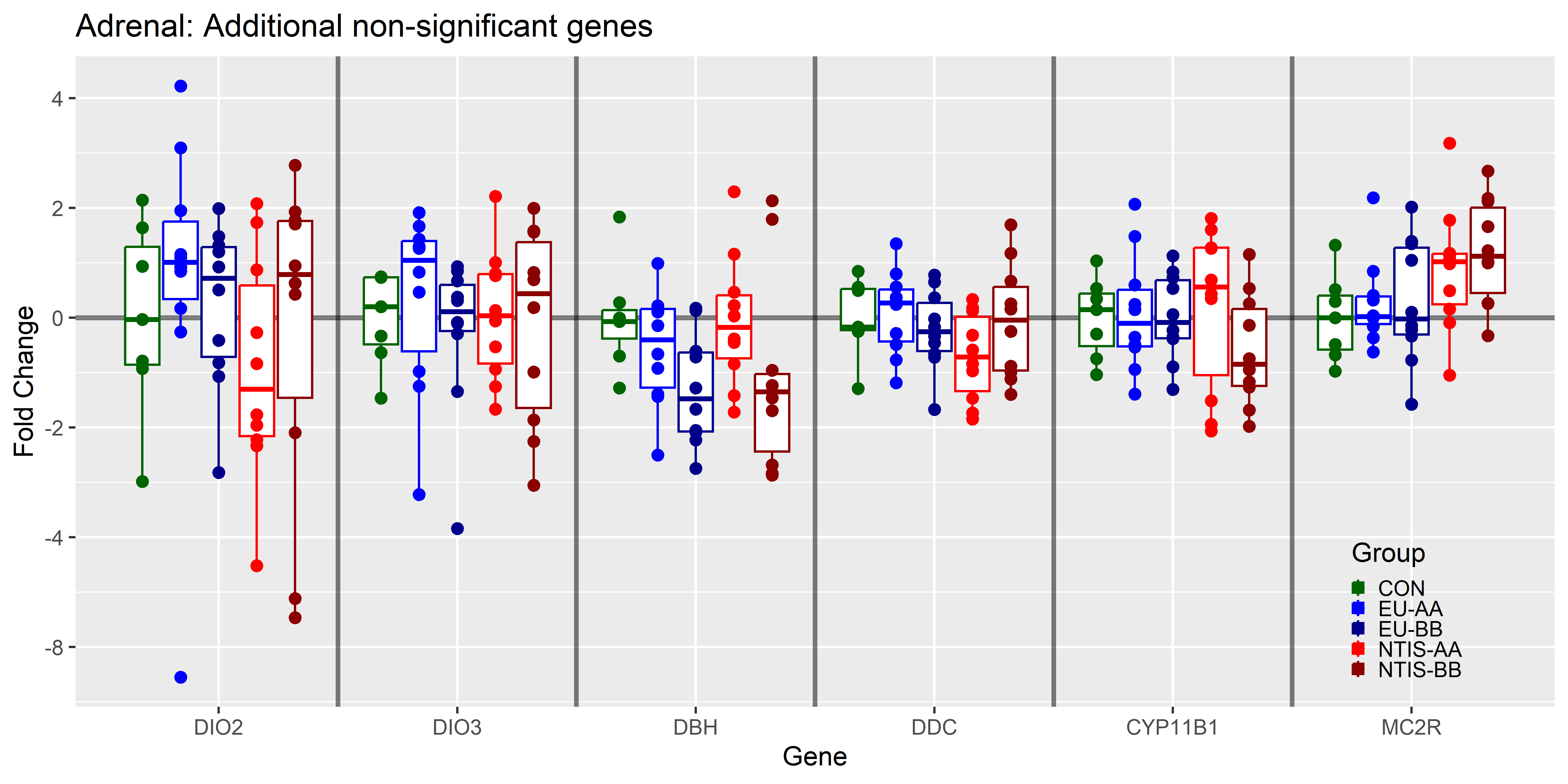

Supplement: Supplementary file 5 — Figure S5: Expression of additional genes assessed in the adrenal gland (ADR) from control (CON), euthyroid uninfected (EU) and infected low thyroid hormone (NTIS) fetuses by rs80998415 genotype (AA or BB). [file CPH4-16-e70112-s003.tiff]
